# Treatment outcomes of tuberculosis patients in nigist Eleni Mohammed general hospital, hosanna, southern nations, nationalities and peoples region, Ethiopia: a five year (June 2009 to August 2014) retrospective study

**DOI:** 10.1186/s13690-017-0184-x

**Published:** 2017-04-03

**Authors:** Tigist Mohammed, Kidist Daniel, Degefa Helamo, Taye Leta

**Affiliations:** 1 Department of Medical Laboratory, Hossana College of Health Sciences, Hosanna, Ethiopia; 2 Department of Clinical Nursing, Hossana College of Health Sciences, Hosanna, Ethiopia; 3 Department of Health Extension, Hossana College of Health Sciences, Hosanna, Ethiopia

**Keywords:** TB, Treatment outcome

## Abstract

**Background:**

Tuberculosis remains to be a major public health problem among under developed world due to delay in detection and treatment of patients with active TB. In Ethiopia, tuberculosis has been recognized as a major public health problem for more than fifty years.

**Objective:**

The main objective of this study was to determine treatment outcomes and associated factors among TB patients attending Nigist Eleni Mohammed General Hospital, Hosanna, SNNPR, Ethiopia.

**Methods:**

A five years medical records on treatment outcomes of tuberculosis was reviewed by using a retrospective study design. A total of 768 tuberculosis patients’ cards registered in TB unit register from June 2009 to August 2014 were reviewed. Data was coded, cleaned and entered into a computer data base by using EPI Info version 3.5.3 and then analysed by using Spss version 20.0 Descriptive summary values such as frequency and percentage was used to describe the study variable. Moreover, bivariate and multivariate logistic regression analysis with a confidence level of 95% was performed in order to determine the final predictors of the outcome variable. Association of age, sex, residence, HIV status of the patient and TB type/category was assessed with the TB treatment outcome through bivariate analysis. And residence, TB category and HIV status were found significantly associated with the treatment outcomes in bivariate analysis. Finally, the forward addition model was used for the multivariate analysis, and residence, TB category and HIV status of TB patient were entered into the final model to obtain an adjusted odds ratio (AOR).

**Result:**

Out of 768 TB patients who were registered at the hospital during the study period, 249 (32.4%) completed the treatment, 84 (10.9%) cured, 11 (1.4%) defaulted, 397 (51.7%) were transferred out to other health facility, 23 (2.9%) died and 4 (0.5%) failed the treatment regimen. In this study, the overall treatment success of TB was 333 (43.3%) as compared to their counterparts, 435 (56.7%). Patients who presented pulmonary TB + ve were more likely to develop risk of poor treatment outcomes as compared to the patients with extra pulmonary TB and pulmonary TB-ve (AOR = 1.915,95% CI;1.213,3.028). The proportion of TB HIV co-infection was16.4%, and HIV + ve TB patients were more likely to develop risk of poor treatment outcomes as compared to their counterparts (AOR = 0.796, 95% CI;0.512,1.236).

**Conclusion:**

From this study, it was generally observed that the rate of defaulting was very low in the hospital. On the other hand, it was observed that the rate of transfer out of patients from the hospital to other health care facilities was very high during the study period. Furthermore, it was observed that patients who came from urban area were less likely to develop risk of poor treatment outcomes as compared to patients who reside in the rural areas.

## Background

Tuberculosis is one of the oldest disease that affect human and the leading cause of death globally. It is caused by Mycobacterium tuberculosis mainly affecting the lungs, but can affect other sites as well and it is curable if properly treated. World Health Organisation (WHO) declared tuberculosis as a global public health emergency by the year 1993 and DOTS program has been promoted as a control strategies [1]. Unlike the availability of an appropriate prevention and control strategies, tuberculosis continues to challenge the world by its incidence and mortality fueled by HIV pandemic and drug resistant TB. The burden of the disease doubles in Africa due to theHIV and a low coverage of HIV testing [2].

The success of TB treatment is the sum of the patients who are cured and those who have completed treatment under the Directly Observed Therapy Short Course (DOTS) strategy. In 1995 Directly Observed Therapy Short Course (DOTS) was initiated for the first time in some areas of Southern Nations Nationalities and Peoples Region and the program has raised the case detection and treatment success rate by folds. However, tuberculosis (TB) remains the leading cause of morbidity and mortality in the region and it is the third cause of inpatient death in the region. Studies conducted in the region revealed that the mortality is higher in the first 2 months of treatment [3].

In the southern region of Ethiopia, tuberculosis was the third cause of death in hospitalized patients. Researches revealed that the mortality rate was 2.5% per annum in successfully treated tuberculosis patients in SNNPR [3].

Although the key target of TB control in DOTs is to detect the disease and treat the cases, delayed presentation for treatment, incomplete treatment or poor compliance as well as treatment interruption or default, relapse, and death are the major challenge that TB programmes face in resource-constrained countries [4].

Moreover, the federal ministry of health (FMOH) reported in 2011 that tuberculosis was the third leading cause of death in Ethiopia. World Health Organisation (WHO) global report in 2012 estimated that there were 8.7 million new cases and 12 million prevalent cases of tuberculosis globally in 2011 [5]. The DOTs strategy, developed by the WHO for the prevention and control of TB in the early 1995s, was believed to be the most valuable strategy; and a cost-effective health intervention for reducing the incidence and death of TB in developing countries [5].

It is estimated that approximately one-third of the world’s population is infected with mycobacterium tuberculosis. Death from active form of tuberculosis is expected to increase to 5 million by the year 2015. The burden the disease will be expected to be much higher in sub-Saharan African countries. The main reasons for the high morbidity and mortality of TB in these regions are dramatic increase in poverty, HIV epidemic, and emergence of drug resistant TB [6].

According to the 2011 national population survey of Ethiopia, there were an estimated 15,000 deaths due to tuberculosis [7].

Ethiopia was ranked 7th in the world for TB burden and 3rd in Africa in 2008, with an estimated TB incidence (all forms) of 378 new cases per 100,000 persons and163 new smear positive cases per 100,000 persons [8].

A study conducted among 6580 registered tuberculosis patients (3147 males and 3433 females) in Addis Ababa health centers showed the following treatment outcomes: 18.1% cured, 64.6% completed treatment, 3.7% died during follow-up, 5.1% defaulted, 0.4% failed the treatment and 8.2% were transferred out to another health institution. From this study, it was found that year of enrollment was significantly associated with the treatment success [8].

A study conducted in Uganda revealed that a limited information of patients about the disease resulted in high rate of defaulting and Mortality. Among 657 TB patients to assess a long term outcome of smear positive TB. Accordingly, 326 (49.6%) interrupted 1or more times. Of which 95 (29.1%) were in intensive phase, 82 (25.2%) in continuation phase and 149 (45.1%) interrupted their treatment in both phases [9]. WHO estimates that 1.9% of all new TB cases may be resistant to the first line anti-tuberculosis drugs while about 9.4% TB cases may be resistance to the previously treated drugs. In patients on anti-tuberculosis drug therapy, poor adherence is recognized as a major cause of treatment failure, relapse and drug resistance [10].

In another study conducted in Ibadan, Nigeria, the following proportion of treatment outcomes were observed: cure (76.6%), failure (8.1%), default (6.6%), transferred out (4.8%), and death (1.9%). The mean age of cured patients was 31.2±3.1 years, which was significantly lower than the mean age of those with poor treatment outcomes. In this study, males had a higher risk of a poor treatment outcome than females. Moreover, patients with a poor knowledge of tuberculosis had a higher risk of having a poor treatment outcome compared to those with a good knowledge about the disease [11].

According to the study conducted in the central part of Ethiopia, the outcome of smear positive pulmonary TB treatment success rate was higher as compared to the WHO targets and showed 10.8% of unfavorable outcome. The unsuccessful treatment outcome was also higher in age groups more than 40, family size>5, retreatment and unemployed compared to their inverse [12].

One study conducted on treatment outcome of tuberculosis patients under directly observed treatment and another five years retrospective study conducted on childhood tuberculosis and its treatment outcomes in Addis Ababa at various times showed almost similar increasing proportions of smear positive PTB, smear negative PTB and EPTB [13, 14].

Treatment outcomes were documented for 95.2% of children of whom 85.5% were successfully treated while rates of mortality and defaulting from treatment was 3.3% and 3.8%, respectively. The proportion of children with TB tested for HIV reached 88.3% during the final year of the study period compared to only 3.9% at the beginning of the study period [14].

Mortality was significantly higher among under-five children (p < 0.001) and those with HIV co-infection (p <0.001). On multivariate logistic regression, children 5–9 years [AOR = 2.50 (95% CI 1.67-3.74)] and 10–14 years [AOR = 2.70 (95% CI 1.86–3.91)] had a significantly higher successful treatment outcomes. On the other hand, smear positive PTB [AOR = 0.44 (95% CI 0.27–0.73), HIV co-infection (AOR = 0.49 (95% CI 0.30–0.80)] and unknown HIV sero-status [AOR = 0.60 (95% CI 0.42-0.86)] were predictors of poor treatment outcomes [14].

From the study conducted in Mizan Aman hospital, it was found that out of 2043 TB patients, male patients outweighed (58.00%) female patients (42.00%). The following treatment outcomes were also observed in this study: 79 (3.87%) cured, 4 (0.20%) defaulted, 1575 (76.99%) transferred out to other health facility, and 25 (1.22%) died [15].

Despite the continued DOTS TB treatment practice in Hadiya zone, treatment outcomes have not been assessed yet in the area. Therefore, the aim of this study was to assess treatment outcomes of TB patients and associated factors in the past five years in Nigist Eleni Mohammed general hospital, Hossana, SNNPR, Ethiopia.

## Methods

### Study area

The study was conducted in Hossana town, Nigist Eleni Mohammed General Hospital (NEMGH). Hossana is capital of Hadiya zone, which is situated 232kms Southwest of Addis Ababa with an average elevation of 2276 m above the sea level. The town has a total area of 23 sq.km (Hossana Municipality). Based on the 2007 census report, its total population was estimated to be 92,733 in 2011 (Zonal report,2007).

### Study design

A retrospective study was conducted from June 2009 to August 2014 among TB patients who were under treatment in the year before August 30/2014 in Nigist Eleni Mohammed general hospital, Hossana.

### Study population

The study population was all patient cards/charts of tuberculosis registered and put on DOTS in the hospital during the study period

#### Inclusion criteria

All patient cards/charts of tuberculosis registered form June 2009 and completed before August 2014.

### Exclusion criteria

Incomplete patient cards were excluded from the study.

### Data collection tool

Medical records were reviewed by using a structured data sheet prepared in English. A treatment outcome was evaluated in accordance with the National Tuberculosis and Leprosy Control Program (NTLCP) adopted from the WHO.

### Data quality assurance

To assure the quality of data, data collectors and supervisors were selected based on their educational status and experience in a TB clinic. After giving a one day’s training for data collectors and supervisors, strict supervision was assumed, mean while any doubts in the questionnaire/checklist was clarified.

### Data processing and data analysis

After data collection, each structured data sheet was checked for its completeness before the data entry. Data was coded, edited and entered into a computer database by using EPI Info version 3.5.3 and then analysed by using Spss version 20.0. Descriptive summary values such as frequency and percentage was used to describe the study variable. Moreover, bivariate and multivariate logistic regression analyses with a confidence level of 95% was performed in order to determine the final predictors of the outcome variable. Variables with *p*-value < 0.05 in the bivariate analysis were transferred into multivariate logistic regression analysis. Finally adjusted odds ratios with their 95% confidence intervals and explanatory variables with p-value of 0.05 were considered to have significant association with the outcome variable.

### Study Variables

#### Dependent variable

TB Treatment out come.

#### Independent variable

Age, Sex, residence, TB type/category, HIV status.

### Ethical consideration

Before the study begins ethical clearance was obtained from ethical and research approval committee of Hossana Collage of Health Sciences. Official permission was secured from authority of the hospital. To maintain confidentiality, names or other identifiers of study participants were not included.

### Limitations of the study

Since the source of data is secondary, the quality issue is always the question leading to certain disorganization of the required information. Because of the incompleteness of some of patient records in this hospital, the study couldn’t address other common risk factors other than the variables included in this study.

### Strengths of the study

This study used structured and standardized data sheet to collect information on TB treatment outcomes. Moreover, this study used only complete patient records to determine and evaluate TB treatment outcomes and associated factors in accordance with the National Tuberculosis and Leprosy Control Program of the WHO. Furthermore, clear-cut recommendations or policy guide was forwarded by this study.

## Result

A total of 768 TB patients were registered at Hossana Nigist Eleni Mohammed general hospital (NEMGH) from June 2009 to August 2014. Of these, 425 (55.3%) were males and 343 (44.7%) were females.

Among the study participants, 208 (27.1%) were in the age group from 25 – 34 years. Out of the total 768 cases, 411 (53.5%) came from rural areas. Regarding TB classifications, about 497 (64.3%) were smear negative pulmonary TB cases.

In terms of patients category 651 (84.8%) were pulmonary TB and 111 (15.2%) were extra pulmonary TB. About 125 (16.5%) of the study participants were HIV positive. TB cases were categorized into new and previously treated, and the majority of them (94.3%) were new cases [Table 1].

**Table 1:** Patient characteristics of study subjects, Nigist Eleni Mohammed General Hospital, June 2009 August 2014

Variable and response category	frequency	Percentage (%)
Sex		
Male	425	55.3
female	343	44.7
Residence		
Urban	353	46.0
rural	415	54.0
Age		
< 14	103	13.4
15-24	194	25.3
25-34	208	27.1
35-44	127	16.5
45-54	70	9.1
55-64	40	5.1
> 65	26	3.4
Tb type		
Smear positive	154	20.1
Smear negative	497	64.7
Extra pulmonary TB	117	15.2
TB category		
Pulmonary TB	651	84.8
Extra pulmonary TB	117	15.2
Treatment category		
New	724	94.3
Previously treated	47	6.1
HIV status		
Positive	126	16.3
Negative	642	83.6

### Treatment Outcomes

Out of 768 TB patients who were registered at the hospital during the study period, 249 (32.4%) completed the treatment, 84 (10.9%) cured, 11 (1.4%) defaulted, 395 (51.7%) were transferred out to other health facility, 22 (2.9%) died and 4 (0.5%) failed the treatment regimen. The treatment success rate (it was computed as: completed the treatment with negative TB bacteria result (cured) plus completed the treatment but without bacteriology result at the end of treatment) was 333 (43.3%) as compared to their counterparts, 435 (56.7%) [Table 2].

**Table 2:** Treatment outcome of study subjects, Nigist Eleni Mohammed General Hospital, June 2009- August 2014

Variable information	frequency	Percentage (%)
Treatment out come for all type of TB		
Cured	84	10.9
Treatment completed	249	32.4
Died	23	3.0
Failed	4	0.5
Transferred out	397	51.7
Defaulted	11	1.4
Overall treatment outcome		
Good treatment outcome	333	43.3
Poor treatment outcome	435	56.7

### Factors associated with treatment outcome of tuberculosis

Association of age, sex, residence, HIV status of the patient and TB type/category was assessed with the TB treatment outcome through bivariate analysis. And residence, TB category and HIV status were found significantly associated with the treatment outcomes in bivariate analysis. Finally, the forward addition model was used for the multivariate analysis, and residence, TB category and HIV status of TB patient were entered into the final model to obtain an adjusted odds ratio (AOR). Those patients who came from urban area were less likely to develop a risk of poor treatment outcomes as compared to patients who reside in rural areas (AOR:0.145; 95% CI :0.104,0.201). Those patients with smear positive (pulmonary TB + ve) were more likely to have poor treatment outcomes as compared to patients with EPTB and smear negative pulmonary TB (AOR = 1.915, 95% CI;1.213,3.028), and HIV positive TB patients were also more likely to develop risk of poor treatment outcomes as compared to their counterparts (AOR = 0.796, 95% CI;0.512,1.236) [Table 3].

**Table 3:** Multivariable logistic regression model predicting treatment outcome, Nigist Eleni Mohammed General Hospital, June 2009 - August 2014

factors	category	Treatment outcome				Crude OR	Adjusted OR
						(95% CI)	(95% CI)
		Good treatment outcome		poor treatment outcome			
		N	%	N	%		
Residence	Rural	92	27.6	323	74.3	0.132 (0.096,0.183)	0.145 (0.104,0.201
	Urban	241	72.4	112	25.7	1	1
TB type(category)	PTB + ve	92	27.6	62	14.3	2.003(1.334,3.007)	1.915(1.213,3.028
	PTB -ve	192	57.7	318	73.1	0.760(0.468,1.234)	0.928(0.538,1.599
	Extra pulmonary TB	62	18.6	55	12.6	1	1
HIV	Positive	69	20.7	56	12.9	0.565(0.384,0.832)	0.796(0.512,1.236
	negative	264	79.3	323	87.1	1	1

## Discussion

In this facility based retrospective study, data was extracted from 768 registered TB patients; and the proportion of male patients (55.3%) dominated female patients. This study was consistent with the finding in Uzbekistan (60%) [16], Turkey (65%) [17], and at Mizan-Aman general hospital (57.2%) [15]. These studies reported an outweighed disease proportion of male patients over female patients. This might be attributed to either males were more likely to develop the disease or more likely to utilize the health services than females. However, one study conducted in Addis Ababa on childhood tuberculosis contradicted this finding.

In this study, out of 768 TB patients, 651 (84.8%) were pulmonary TB patients, and 117 (15.2%) were extra pulmonary TB patients. And among the pulmonary TB patients who were registered during the study period, the majority were affected by smear negative pulmonary TB (64.7%). And this finding was almost similar with the study finding conducted in Uzbekistan. In this study, pulmonary TB (PTB) was present in 77%, of which 43% were smear-positive and 53% were smear-negative [16], with the study finding conducted at Mizan- Aman general hospital (51.05%) [15], and This finding was also supported with the study finding conducted in Kwekwe district, Zimbabwe. In this study 42.4% of patients were with pulmonary tuberculosis and 8.2% of patients were with extra pulmonary tuberculosis. And among the pulmonary TB patients who were registered during the study period, the majority were affected by smear negative pulmonary TB (64.7%) [18].

As to TB HIV co-infection; this study reported 16.4% TB HIV co-infection which was in line with the Ethiopian federal ministry of health report by 2009/10 (15%), and with the finding observed in the study conducted at Mizan-Aman general hospital (16.5%) [2, 15]. In this study, HIV+ve TB patients were more likely to develop risk of poor treatment outcomes as compared to HIV-ve TB patients (AOR=0.796, 95% CI;0.512,1.236). This finding was almost in line with the study conducted in a cohort of tuberculosis patients in Recife, Pernambuco state, Brazil and showed that HIV+ve TB patients had developed poor treatment outcomes as compared to their counterparts (AOR=3.19, 95% CI; 1.31,7.73), and with the finding reported from the study conducted in Kwekwe district, Zimbabwe (RR=2.07, 95% CI;1.12,3.81). Another [18, 19].

In this study, the overall proportion of cured patients was 10.9%, which was higher than the proportion observed at the study conducted in Mizan-Aman general hospital (3.87%) [15]. The difference might be explained due to the high transfer out observed in Mizan-Aman general hospital (76.99%). In this study, the cure rate of TB smear positive was 79.39%, which was higher as compared with the study reported in Addis Ababa (64.8%) and Mizan-Aman general hospital (62.7%) [14, 15]. This difference might be due to a better access to health care services and information in the latter settings.

The defaulting rate in this study (1.4%) revealed lower finding than the previous studies conducted in Nigist Eleni general hospital (20%), in Uzbekistan (6%), in Turkey (3.9%), in hospitals in Imo state, Nigeria (9.8%), and in Gondar University teaching hospital (36.4%), [16, 17, 20, 21]. This lower defaulting rate in this study might be due to a better supervision and health education activities than the previous study areas.

The overall proportion of death (2.9%) in this study was lower than the death rate reported in the study conducted in Dessie and Woldiya town health institutions, Northeast Ethiopia (8.1%), in hospitals in Imo state, Nigeria (6.5%), in Addis Ababa (4%), in Felege Hiwot referral hospital, Northwest Ethiopia (5.8%), and in Kwekwe district, Zimbabwe (8.7%) [14, 18, 20, 22, 23]. The observable higher death rate in the above study areas might be due to the lack of strict follow up and defaulter tracing mechanism. However, the overall death rate observed in this study was higher than the death rate observed in Turkey (2.4%) and in Recife, Pernambuco state, Brazil (2.8%) [17, 19].

Furthermore, this study reported a treatment failure rate of 0.5%, which was consistent with the rate of treatment failures reported in Enfraz health center (0.5%) and in Felege Hiwot referral hospital, Northwest Ethiopia (0.5%) [23, 24]. However, the treatment failure reported in this study was slightly higher than the finding in different health centers in Kotabharu, Kelantan, Malaysia (0.2%), and in Addis Ababa (0.4%) [13, 25]. On the other hand, the treatment failure observed in this study was lower than the study findings observed in: Dessie and Woldiya town health institutions, Northeast Ethiopia (0.8%), Uzbekistan (3%), Turkey (1.1%), Recife, Pernambuco state, Brazil (2.1%), hospitals in Imo state, Nigeria (1.5%), and Kwekwe district, Zimbabwe (0.9%) [16–20, 22].

There was also a high transfer out rate (51.7%) observed in this study among the patients who registered for the TB treatment. But this finding was lower than the study finding in Felege Hiwot referral hospital, Northwest Ethiopia (68.6%) [23]. This high rate might be due to the overflow of patients from the rural areas (53.5%). However, the transferred out cases might have been cured or successfully completed the treatment. It could also happen due to unfavorable TB treatment outcomes like treatment failure and default unless adequate care and information is considered to the patients. This means, lack of information about transferred out cases therefore, limits the strength of reports about treatment success rate from DOTS clinics.

The overall treatment success of TB was 43.3%, which was high as compared to the previous finding in Felege Hiwot referral hospital, Northwest Ethiopia (26%) [23], but very low as compared to the findings in: Dessie and Woldiya town health institutions, Northeast Ethiopia (88.1%), Uzbekistan (83%), Turkey (92.6%), Recife, Pernambuco state, Brazil (70.1%), Kotabharu, Kelantan, Malaysia (93%), hospitals in Imo state, Nigeria (81.4%), and Kwekwe district, Zimbabwe (72.4%) [16–20, 22, 25]. Possible elucidations for the observed differences between the findings might be explained due to a high and/or low transfer out rate in each of the study area.

In this study, residence and treatment outcomes were highly associated. This means those patients who came from rural areas were more likely to develop risk of poor treatment outcomes as compared to patients who reside in urban areas (AOR=0.145 95% CI; 0.104,0.201). This finding was parallel with the study findings in: Uzbekistan (AOR=1.3,95% CI;1.2,1.4), Felege Hiwot referral hospital, Northwest Ethiopia (RR =7.0, 95% CI;3.89,12.63), and Kwekwe district, Zimbabwe (AOR=1.91, 95% CI;1.14,3.20) [16, 18, 23].

This might be due to better health seeking behavior of patients living in the urban areas. Moreover, the health care institutions might be located nearby the patients living in urban areas, which in turn might contribute for a better health seeking behavior and treatment outcomes.

In this study, there was also a significant association between treatment out come and TB category. This means patients who presented pulmonary TB+ve were more likely to develop risk of poor treatment outcomes as compared to the patients with extra pulmonary TB and pulmonary TB-ve (AOR=1.915,95% CI;1.213,3.028). This finding contradicted the finding that says no significant association between unsuccessful treatment out comes and a status of pulmonary TB patient as reported in the study finding conducted in Dessie and Woldiya town health institutions, Northeast Ethiopia (AOR= 0.58, 95% CI; 0.29,1.14), in Enfraz health center, North west part of Ethiopia, and in Felege Hiwot referral hospital, Northwest Ethiopia (AOR= 1.4, 95% CI; 1.13,1.84). In these studies, the risk of developing unsuccessful treatment outcomes was less likely among the pulmonary TB patients than their counterparts [22–24]. This might happen due to the proportion of TB patients considered in each category for the study.

In this facility based retrospective study, data was extracted from 768 registered TB patients; and the proportion of male patients (55.3%) dominated female patients. This study was consistent with the finding at Mizan-Aman general hospital (57.2%), Turkey (65%), and Uzbekistan (60%). [15–17]. These studies reported an outweighed disease proportion of male patients over female patients. This might be attributed to either males were more likely to develop the disease or more likely to utilize the health services than females. However, one study conducted in Addis Ababa on childhood tuberculosis contradicted this finding. The study showed a high number of EPTB proportion among female patients [14].

In this study, out of 768 TB patients, 651 (84.8%) were pulmonary TB patients, and 117 (15.2%) were extra pulmonary TB patients. This finding was supported with the study finding conducted in Kwekwe district, Zimbabwe. In this study 42.4% of patients were pulmonary tuberculosis and 8.2% of patients were extra pulmonary tuberculosis. Among the pulmonary TB patients who were registered during the study period, the majority were affected by smear negative pulmonary TB (64.7%). And this finding was almost similar with the study finding conducted at Mizan- Aman general hospital (51.05%) [15], and with the study finding conducted at Uzbekistan. In this study, pulmonary TB (PTB) was present in 77%, of which 43% were smear-positive and 53% were smear-negative [16].

As to TB HIV co-infection; this study reported 16.4% TB HIV co-infection which was in line with the Ethiopian federal ministry of health report by 2009/10 (15%) [2], and with the finding observed in the study conducted at Mizan-Aman general hospital (16.5%) [15]. In this study, HIV + ve TB patients were more likely to develop risk of poor treatment outcomes as compared to HIV-ve TB patients (AOR = 0.796, 95% CI;0.512,1.236). This finding was almost in line with the finding reported from the study conducted in Kwekwe district, Zimbabwe (RR = 2.07, 95% CI; 1.12, 3.81). Another study conducted in a cohort of tuberculosis patients in Recife, Pernambuco state, Brazil showed that HIV + ve TB patients had developed poor treatment outcomes as compared to their counterparts (AOR = 3.19, 95% CI; 1.31,7.73) [18, 19].

In this study, the overall proportion of cured patients was 10.9%, which was higher than the proportion observed at the study conducted in Mizan-Aman general hospital (3.87%). The difference might be explained due to the high transfer out observed in Mizan-Aman general hospital (76.99%). In this study, the cure rate of TB smear positive was 79.39%, which was higher as compared with the study reported in Addis Ababa (64.8%) and Mizan-Aman general hospital (62.7%) [14, 15]. This difference might be due to a better access to health care services and information in the latter settings.

The defaulting rate in this study (1.4%) revealed lower finding than the previous studies conducted in Nigist Eleni general hospital (20%), in Gondar University teaching hospital (36.4%) (24), in Turkey (3.9%), in Uzbekistan (6%), and in hospitals in Imo state, Nigeria (9.8%) [16, 17, 20, 21]. This lower defaulting rate in this study might be due to a better supervision and health education activities than the previous study areas.

The overall proportion of death (2.9%) in this study was lower than the death rate reported in the study conducted in Addis Ababa (4%), in Felege Hiwot referral hospital, Northwest Ethiopia (5.8%), in Dessie and Woldiya town health institutions, Northeast Ethiopia (8.1%), in hospitals in Imo state, Nigeria (6.5%), and in Kwekwe district, Zimbabwe (8.7%) [12, 14, 18, 20, 22]. The observable higher death rate in the above study areas might be due to the lack of strict follow up and defaulter tracing mechanism. However, the overall death rate observed in this study was higher than the death rate observed in Turkey (2.4%) and in Recife, Pernambuco state, Brazil (2.8%) [17, 19].

Furthermore, this study reported a treatment failure rate of 0.5%, which was consistent with the rate of treatment failures reported in Enfraz health center (0.5%) and in Felege Hiwot referral hospital, Northwest Ethiopia (0.5%) [12, 24]. However, the treatment failure reported in this study was slightly higher than the finding in different health centers in Addis Ababa (0.4%), and in Kotabharu, Kelantan, Malaysia (0.2%) [13, 25]. On the other hand, the treatment failure observed in this study was lower than the study findings observed in: Dessie and Woldiya town health institutions, Northeast Ethiopia (0.8%), Uzbekistan (3%), Turkey (1.1%), Recife, Pernambuco state, Brazil (2.1%), hospitals in Imo state, Nigeria (1.5%), and Kwekwe district, Zimbabwe (0.9%) [16–20, 22].

There was also a high transfer out rate (51.7%) observed in this study among the patients who registered for the TB treatment. But this finding was lower than the study finding in Felege Hiwot referral hospital, Northwest Ethiopia (68.6%) [12]. This high rate might be due to the overflow of patients from the rural areas (53.5%). However, the transferred out cases might have been cured or successfully completed the treatment. It could also happen due to unfavorable TB treatment outcomes like treatment failure and default unless adequate care and information is considered to the patients. This means, lack of information about transferred out cases therefore, limits the strength of reports about treatment success rate from DOTS clinics.

The overall treatment success of TB was 43.3%, which was high as compared to the previous finding in Felege Hiwot referral hospital, Northwest Ethiopia (26%) [12], but very low as compared to the findings in: Dessie and Woldiya town health institutions, Northeast Ethiopia (88.1%), Uzbekistan (83%), Turkey (92.6%), Recife, Pernambuco state, Brazil (70.1%), Kwekwe district, Zimbabwe (72.4%), hospitals in Imo state, Nigeria (81.4%), and Kotabharu, Kelantan, Malaysia (93%) [16–20, 22, 25]. Possible elucidations for the observed differences between the findings might be explained due to a high and/or low transfer out rate in each of the study area.

In this study, residence and treatment outcomes were highly associated. This means those patients who came from rural areas were more likely to develop risk of poor treatment outcomes as compared to patients who reside in urban areas (AOR = 0.145 95% CI; 0.104,0.201). This finding was parallel with the study findings in: Felege Hiwot referral hospital, Northwest Ethiopia (RR = 7.0, 95% CI;3.89,12.63), Uzbekistan (AOR = 1.3,95% CI;1.2,1.4), and Kwekwe district, Zimbabwe (AOR = 1.91, 95% CI;1.14,3.20) [12, 16, 18].

This might be due to better health seeking behavior of patients living in the urban areas. Moreover, the health care institutions might be located nearby the patients living in urban areas, which in turn might contribute for a better health seeking behavior and treatment outcomes.

In this study, there was also a significant association between treatment out come and TB category. This means patients who presented pulmonary TB + ve were more likely to develop risk of poor treatment outcomes as compared to the patients with extra pulmonary TB and pulmonary TB-ve (AOR = 1.915,95% CI;1.213,3.028). This finding was in opposite to the study finding conducted in Dessie and Woldiya town health institutions, Northeast Ethiopia (AOR = 0.58, 95% CI; 0.29,1.14), and in Felege Hiwot referral hospital, Northwest Ethiopia (AOR = 1.4, 95% CI; 1.13,1.84). In these studies, the risk of developing unsuccessful treatment outcomes was less likely among the pulmonary TB patients than the extra pulmonary TB patients [12, 22]. This might happen due to the proportion of TB patients considered in each category for the study. On the other hand, patients with smear positive (pulmonary TB + ve) were more likely to develop a risk of poor treatment outcomes as compared to smear negative (pulmonary TB-ve) patients (AOR = 1.915 95% CI;1.213,3.028). This result contradicted the finding that says no significant association between unsuccessful treatment out comes and a status of pulmonary TB patient as reported in Enfraz health center in North west part of Ethiopia [24].

## Conclusion

From this study it was generally observed that the rate of defaulters was very low in the hospital. On the other hand, it was observed that the rate of transfer out of patients from the hospital to other health care facilities was very high during the study period.

TB classification, residence and HIV status of the patients were found significantly associated with TB treatment outcomes, and patients who came from urban area were less likely to come up with poor treatment outcome as compared to patients who reside in rural areas.

Furthermore, it was observed that those patients with pulmonary smear positive (pulmonary TB + ve) and those with the history of HIV (HIV + ve) were more likely to develop risk of poor treatment outcomes as compared to their counterparts.

### Recommendations

#### To zonal health department and NEMGH

During the study period there was a significant number of patient transfer out. Therefore, based on this finding, it is better for the hospital and zonal health department to carry out a strict and frequent supervision to access the relevant information regarding the treatment outcome of the patients from the accepting health care facilities.

From the study, it was observed that there was poor treatment outcomes among the patients coming from the rural areas. Therefore, the hospital and zonal health department has to enable the rural population with educational, environmental, organisational and other relevant facilities, so that the health seeking behavior of the population towards TB will be improved.

## Abbreviations

CDR: Crude death rate
DOTs: Direct observed treatment strategies
ERA: Ethical and research approval
FMOH: Federal ministry of health
HIV: Human immune deficiency virus
MTB: Mycobacterium tuberculosis
NEMGH: Nigist eleni mohammed general hospital
NTLCP: National tuberculosis and leprosy control program
PORT: Patient outcomes research team
PTB: Pulmonary tuberculosis
RRT: Research review technical
SCC: Short-course chemotherapy
SNNPR: Southern nations, nationalities and peoples region
SPSS: Statistical package for social science
TB: Tuberculosis
TSR: Treatment success rate
WHO: World Health Organization
